# Understanding the riddle of amine oxidase flavoenzyme reactivity on the stereoisomers of *N*‐methyl‐dopa and *N*‐methyl‐tyrosine

**DOI:** 10.1002/jmr.3068

**Published:** 2023-11-15

**Authors:** Oriol Gracia Carmona, Majd Lahham, Peter Poliak, Dominic Goj, Eva Frießer, Silvia Wallner, Peter Macheroux, Chris Oostenbrink

**Affiliations:** ^1^ Institute for Molecular Modeling and Simulation, Department of Material Sciences and Process Engineering University of Natural Resources and Life Sciences Vienna Austria; ^2^ Institute of Biochemistry Graz University of Technology Graz Austria; ^3^ Department of Biochemistry and Microbiology, Faculty of Pharmacy Arab University for Science and Technology Hama Syria; ^4^ Christian Doppler Laboratory Molecular Informatic in the Biosciences University of Natural Resources and Life Sciences Vienna Austria

**Keywords:** free‐energy calculations, FsqB, GROMOS, molecular dynamics simulations, molecular recognition, site‐directed mutagenesis

## Abstract

Enzymes are usually stereospecific against chiral substrates, which is commonly accepted for the amine oxidase family of enzymes as well. However, the FsqB (fumisoquin biosynthesis gene B) enzyme that belongs to the family of sarcosine oxidase and oxidizes L‐*N*‐methyl‐amino acids, shows surprising activity for both enantiomers of *N*‐methyl‐dopa. The aim of this study is to understand the mechanism behind this behavior. Primary docking experiments showed that tyrosine and aspartate residues (121 and 315 respectively) are located on the ceiling of the active site of FsqB and may play a role in fixing the *N*‐methyl‐dopa via its catechol moiety and allowing both stereoisomers of this substrate to be in close proximity of the N5 atom of the isoalloxazine ring of the cofactor. Three experimental approaches were used to prove this hypothesis which are: (1) studying the oxidative ability of the variants Y121F and D315A on *N*‐methyl‐dopa substrates in comparison with *N*‐methyl‐tyrosine substrates; (2) studying the FsqB WT and variants catalyzed biotransformation via high‐performance liquid chromatography (HPLC); (3) molecular dynamics simulations to characterize the underlying mechanisms of the molecular recognition. First, we found that the chemical characteristics of the catechol moiety of *N*‐methyl‐dopa are important to explain the differences between *N*‐methyl‐dopa and *N*‐methyl‐tyrosine. Furthermore, we found that Y121 and D315 are specific in FsqB and not found in the model enzyme sarcosine oxidase. The on‐bench and theoretical mutagenesis studies show that Y121 residue has a major role in fixing the *N*‐methyl‐dopa substrates close to the N5 atom of the isoalloxazine ring of the cofactor. Simultaneously, D315 has a supportive role in this mechanism. Jointly, the experimental and theoretical approaches help to solve the riddle of FsqB amine oxidase substrate specificity.

## 1 INTRODUCTION

**FIGURE 1:**
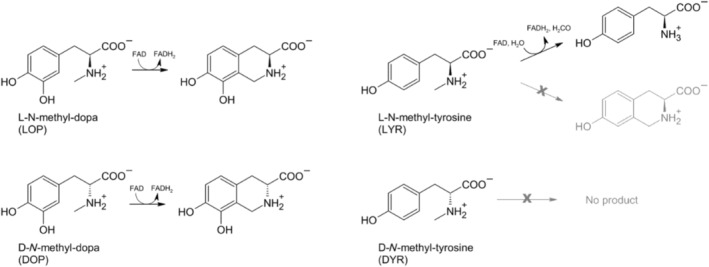
Non‐natural substrates and their products used for studies on FsqB. For *N*‐methyl‐dopa, FsqB is able to catalyze the oxidative cyclization of both enantiomers. For *N*‐methyl‐tyrosine FsqB accepts only the L enantiomer and catalyzes the oxidative demethylation reaction.^4^

FsqB (fumisoquin biosynthesis gene B) is a flavoprotein oxidase belonging to the family of sarcosine oxidases (SOX).^1^ It contains a flavin adenine dinucleotide (FAD) cofactor, which is covalently attached to the enzyme via a cysteinyl linkage. Originally, the enzyme was found in the fungal pathogen *Aspergillus fumigatus*, where it is involved in the biosynthesis of the isoquinoline alkaloid fumisoquin.^2^ FsqB catalyzes the oxidative cyclization of a pathway intermediate containing a catechol moiety. In order to understand the oxidative cyclization mechanism, FsqB was biochemically and structurally characterized in a previous study.^3^ Kinetic parameters of FsqB were determined using non‐natural substrates, most notably *N*‐methyl‐dopa and *N*‐methyl‐tyrosine. Despite the similarity between these substrates (for structures see Figure 1 and see Table 1 for abbreviations), FsqB turned out to be stereospecific for *N*‐methyl‐tyrosine, whereas it completely lost its stereospecificity with *N*‐methyl‐dopa. Therefore, we hypothesize that the catechol moiety of *N*‐methyl‐dopa is crucial for this behavior. Docking scenarios showed that two residues, tyrosine 121 and aspartate 315 fix the ligand by forming hydrogen bonds with the hydroxyl functional groups of the substrate. This type of binding allows the enzyme to fix the catechol moiety of both *N*‐methyl‐dopa enantiomers and hence leads to the oxidation of both L‐ and D‐*N*‐methyl‐dopa. In this study, both on‐bench experiments and molecular dynamic (MD) simulations were applied to prove this hypothesis.

**TABLE 1:** List of ligands and abbreviations.

Name of ligand	Abbreviation
L‐*N*‐methyl‐dopa	LOP
D‐*N*‐methyl‐dopa	DOP
*rac*‐*N*‐methyl‐dopa	ROP
L‐*N*‐methyl‐tyrosine	LYR
D‐*N*‐methyl‐tyrosine	DYR
Sarcosine (*N*‐methyl‐glycine)	SAR

## 2 METHODS

### 2.1 Molecular cloning and site‐directed mutagenesis

The gene coding for FsqB was equipped with a C‐terminal octa‐histidine tag, flanked with *NdeI* and *NotI* restriction sites, and optimized for expression in *Escherichia coli* using the online optimization tool of GeneArt (Thermo Fisher Scientific, Waltham, MA, US). The synthetic gene was cloned into the pET21a expression vector using the aforementioned restriction sites. *E. coli* Top 10 and *E. coli* BL21star cells (both strains produced by Stratagen, Houston, TX, USA) were transformed with the generated expression plasmids, and selection for positive transformants was done using 100 μg/mL ampicillin.

Additionally, site‐directed mutagenesis was applied to generate variant proteins with amino acid replacements Y121F and D315A, respectively. Therefore, wild‐type *fsqb* was used as a template and the primers were designed to produce the desired mutations for the PCR‐based mutagenesis (a list of primer pairs can be found in Table S1). The sequence of all generated plasmids was confirmed by sequencing.

### 2.2 Protein production and purification

The wild‐type protein of FsqB and its variants were produced in shake flasks in an HT Multitron standard shaking system (Infors AG, Basel, Switzerland). One liter of LB medium containing 100 μg/mL ampicillin in plastic flasks was inoculated with fresh preculture to a start optical density of 0.1 at 600 nm. The cultures were incubated at 37°C and 140 rpm until an optical density of 0.7 was reached. Then protein production was induced by adding 300 μM of isopropyl β‐D‐thiogalactopyranoside (IPTG), and the cultures were incubated at 18°C overnight to increase protein yield. The protein‐containing bacterial cells were harvested by centrifugation at 5000 × *g* for 15 min. The enzyme was purified at 4°C. The cells were suspended in lysis buffer (100 mM phosphate/NaOH, 150 mM sodium chloride, 10 mM imidazole, pH 7.6, containing 1 mM phenylmethylsulfonyl fluoride and 0.1 mg/mL lysozyme) and sonicated for 10 min by using an ultrasonic probe (Labsonic U, B. Braun Biotech, Berlin, Germany). The lysate was centrifuged at 38500 × *g* for 60 min. The supernatant was filtered and then loaded on a Nickel‐NTA column (Nickel–nitrilotriacetic acid–Sepharose fast flow column‐GE Healthcare, Chicago, IL, US) preequilibrated with lysis buffer. After loading the lysate, the column was washed with 10 column volumes of washing buffer (100 mM phosphate/NaOH, 150 mM NaCl, 30 mM imidazole, pH 7.6). Then the protein of interest was eluted with elution buffer (100 mM phosphate/NaOH, 150 mM NaCl, 150 mM imidazole, pH 7.6). Protein‐containing fractions were pooled and dialyzed against storage buffer (100 mM phosphate/NaOH, 150 mM NaCl, pH 7.6) overnight to get rid of imidazole salts. The subunit molecular mass and purity of the protein preparation were confirmed by SDS polyacrylamide gel electrophoresis using a 12.5% gel. Proteins were concentrated with ultracentrifugation using Amicon Ultra‐15 centrifugal filters with a cutoff of 30 kDa (Merck – Millipore, Burlington, MA, US), flash‐frozen in liquid nitrogen and stored at −80°C for further use.

### 2.3 Determination of thermal stability

The melting points of FsqB wild‐type and variant proteins were measured to assess the thermal stability using the Thermofluor method. Briefly, the proteins were mixed with SyproOrange as a fluorescent dye and heated in a real‐time PCR device (CFX Connect, Biorad, Hercules, CA, US), where the increase in fluorescence due to protein unfolding was monitored.^4^ All measurements were performed in triplicate with a total volume of 25 μL containing 3 mg/mL protein and 5 μL 5000X SYPRO® Orange Protein Stain (1:200) in 100 mM phosphate/NaOH with 150 mM NaCl (pH 7.6). The temperature program started at 20°C for 5 min, then the temperature was gradually increased in 0.5°C steps every minute until a final temperature of 95°C was reached. The CFX Manager 3.0 software was used to determine the melting temperatures for FsqB wild‐type and variant proteins.

### 2.4 Product identification

Products of enzymatic turnover were identified using HPLC‐MS analysis. Reaction mixtures (V = 1 mL) contained 5 μM enzymes with 5 mM of substrate/ligand in 100 mM sodium phosphate buffer, pH 7.6. Reactions were left for 1 h at 25°C with shaking at 600 rpm. Then the reactions were stopped by adding 200 μL of 2 M guanidine hydrochloride to the mixtures and denatured protein was removed by spinning at 10000 × *g* for 10 min. The supernatant was filtered and analyzed. Low‐resolution mass spectra were measured with an Agilent Technologies 6120 Quadrupole LC/MS detector in combination with an Agilent Technologies 1260 Infinity HPLC system, equipped with a Kinetex 2.6 μM C‐18100 Å column (50 × 4.6 mm, 2.6 μm). An isocratic protocol with water/acetonitrile (0.1 vol% of formic acid) was used for elution.

### 2.5 UV–visible absorption spectroscopy

The UV–vis absorption spectrum of the covalently bound FAD cofactor was measured with a Specord 210 spectrophotometer (Analytik Jena, Jena, Germany) to reveal changes in the flavin spectrum of generated variant proteins and to monitor possible spectral changes caused by ligand binding to the protein. The FAD spectra of wild‐type FsqB and studied variant proteins were measured in free form and in the presence of a 10‐fold excess of the tested ligands. The reduction of flavin was taken as proof of ligand binding.

### 2.6 Computational methods

#### 2.6.1 DFT calculations

All ligand structures and charge distributions were calculated with the Gaussian 16 software package (G16)^5^ and the ωB97X‐D functional.^6^ All calculations were performed in the 6‐311++G(d,p) basis set.^7^, ^8^ The effect of an aqueous environment was included implicitly by the SMD variant of the integral‐equation‐formalism polarizable continuum model.^9^ True local minima were confirmed by the vibrational frequency analysis with no imaginary frequencies. Partial atomic charges were fitted to the molecular electrostatic potential by the Merz–Singh–Kollman scheme.^10^, ^11^

#### 2.6.2 System preparation

The four substrates under study, D‐*N*‐methyl‐dopa (DOP), L‐*N*‐methyl‐dopa (LOP), D‐*N*‐methyl‐tyrosine (DYR), L‐*N*‐methyl‐tyrosine (LYR), were parametrized in their zwitterionic form using the GROMOS 54A8 tyrosine parameters as a Reference 12 and compared to the charges obtained from the DFT/ωB97X‐D/6‐311++G(d,p) calculation. Initial MD simulations of the ligands were compared to quantum‐mechanical (QM) calculations using the B3LYP functional^13^, ^14^ with the D3 version of Grimme's dispersion with Becke–Johnson damping,^15^, ^16^ to validate the internal hydrogen bonding pattern of the vicinal hydroxyl groups. To ensure agreement, additional Lennard‐Jones parameters were added to the hydrogens of the hydroxyl groups of the catechol moiety (see Figure S1).

The FAD cofactor was parametrized in its oxidized form using the GROMOS 54C8 parameters. The FAD cofactor was then covalently bound to CYS 414. All protein force field parameters were taken from the GROMOS 54A8 parameter set,^12^ while water was modeled according to the SPC water model.^17^

The starting structure for the FsqB enzyme was obtained from the crystal structure with PDB code 6GG2.^3^ The ligand DOP was placed in the active site based on the binding pose of sarcosine (*N*‐methyl‐glycine) in the monomeric sarcosine oxidase (PDB code 3QSE), by aligning it to the FsqB crystal structure using Pymol.^3^, ^18^

All relevant files to reproduce the molecular simulations are available in the Supplementary Information.

#### 2.6.3 MD simulations

All the systems under study were first minimized using the steepest‐descent algorithm in vacuum. The systems were then solvated in a rectangular box using SPC water molecules,^17^ leaving a minimum distance of 1.4 nm from the solute to the box walls. To accommodate the solvent, an additional minimization with position restraints on all the solute atoms was performed. After minimization, counter ions were added to the systems to achieve a net charge of 0. This was done by replacing the waters with the highest electronegative potential using the GROMOS++ program ion.^19^ The systems were then thermalized at constant volume and a constant temperature of 300 K using the weak‐coupling thermostat^20^ in five 10 ps simulations, where the temperature was raised progressively. The initial velocities were sampled from a Maxwell‐Boltzmann distribution at 60 K and the thermostat increased by 60 K after each thermalization run. Position restraints were used during thermalization starting from 2.5 × 10^4^ kJ/mol/nm^2^ and decreased by a factor of 10 after each simulation. After thermalization, the pressure was kept constant at 1 atm using the weak coupling algorithm using a relaxation time of 0.5 ps and an estimated isothermal compressibility of 4.575 × 10^−4^ (kJ/mol/nm^3^)^−1^.^20^ Subsequently, the thermostat was switched to Nosé–Hoover‐chains with five chains and relaxation times of 0.1 ps.^21^ The solute and solvent degrees of freedom were coupled to different heat baths. The systems were then equilibrated using this setup, for an additional 1 ns in the case of the ligands in solvent and 10 ns for the protein systems. All the simulations were performed using the GROMOS MD engine.^22^ Newton's equations of motion were solved using a leapfrog integration scheme with a time‐step of 2 fs. The covalent bond lengths of solute and solvent molecules as well as the angles of solvent molecules were constrained at their optimal values using the SHAKE algorithm.^23^ Nonbonded interactions were calculated using a triple‐range cutoff scheme. Interactions within a short‐range cutoff of 0.8 nm were calculated at every time step using a pairlist that was generated every 10 fs. Long‐range interactions within a cutoff of 1.4 nm were calculated at pairlist generation and kept constant between updates. To account for electrostatic interactions beyond the 1.4 nm cutoff, a generalized reaction field^24^ with a relative dielectric permittivity of 61^25^ was used.

#### 2.6.4 Free energy perturbation

Binding free energies were calculated along thermodynamic cycles in which alchemical perturbations were performed twice: one for the ligand bound to the protein and one for the ligand in solvent. The free energy perturbations were performed using the extended TI methodology^26^ and a single topology approach.^27^ The softness parameters were set to *α*_LJ_ = 0.5 and *α*_CRF_ = 0.5 nm^2^ for the atoms being perturbed, and the number of precalculated λ‐points was set to 101. A total of 21 λ‐values were simulated for 2 ns each for the perturbations involving an improper dihedral change and 11 λ‐values for the rest of the perturbations. After obtaining the final binding poses for each of the ligands under study, 40 ns of the simulation were performed additionally for every ligand. The first 20 ns were discarded and the remaining 20 ns were analyzed using the GROMOS++ program suite.^19^

#### 2.6.5 In silico generation of protein variants

Protein variants were generated by progressively transforming the wild‐type amino acids to their mutated version using the GROMOS perturbation code. A total of 11 λ‐values of 1 ns each were performed. The final poses were then simulated for additional 40 ns of conventional MD. The first 20 ns were discarded as equilibration and the remaining 20 ns were analyzed using the GROMOS++ program suite.^19^

## 3 RESULTS

### 3.1 Production, purification, and characterization of FsqB wildtype, Y121F, and D315A variant

Expression plasmids for FsqB wild‐type and variants were generated using standard molecular biology techniques and all generated plasmids were verified by sequencing. The *fsqb* containing plasmids were transformed into *E. coli* expression strains and the proteins were produced using a standard protocol for heterologous gene expression. Produced proteins were purified by nitrilotriacetic acid affinity chromatography. UV–visible absorption spectra were recorded for all studied proteins. The thermal stability of wild‐type FsqB and the generated variants was assessed to confirm that the substitution of one amino acid does not affect the structural integrity of the proteins. Therefore, Thermofluor experiments were performed to determine and compare the melting temperature of the proteins in the storage buffer. The experiments showed that the melting temperatures of the two generated variants were close to the melting temperature determined for the wild‐type protein and thus the variants can be regarded as stable (see Table S2).

### 3.2 FsqB catalyzed biotransformation

In a former study, it was already shown that FsqB is able to accept both stereoisomers of *N*‐methyl‐dopa and only the L‐enantiomer of *N*‐methyl‐tyrosine and that either a cyclization reaction or demethylation of the substrate occurs, respectively.^3^ The reactivity of rac‐*N*‐methyl‐dopa (ROP) is very similar to the reactivity of LOP, indirectly confirming that DOP is a suitable substrate. Since tyrosine 121 and aspartate 315 are most likely responsible for substrate binding and positioning in the active site, biotransformation reactions with FsqB Y121F and D315A were performed and analyzed. It is obvious that the substitution of tyrosine 121 with phenylalanine has a drastic effect on the conversion rate and that tyrosine 121 is crucial for the enzymatic activity of FsqB with these substrates, whereas the substitution D315A moderately affected the conversion rate of the screened substrates, with conversion rates between 28% and 48% (Table 2). It is noteworthy mentioning that both investigated variant proteins did not affect the type of product formed, that is, ring‐closed products and demethylated products were detected for *N*‐methyl‐dopa and *N*‐methyl‐tyrosine, respectively.

**TABLE 2:** Conversion of FsqB catalyzed biotransformation.

Enzyme	Substrate	% of conversion	
		HPLC‐UV	HPLC‐MS
WT	*rac‐N*‐methyl‐dopa	95^a^	76^a^
	L‐*N*‐methyl‐dopa	89^a^	85^a^
	L‐*N*‐methyl‐tyrosine	97^b^	99^b^
	D‐*N*‐methyl‐tyrosine	<1^b^	<1^b^
Y121F	*rac‐N*‐methyl‐dopa	7^a^	5^a^
	L‐*N*‐methyl‐dopa	7^a^	7^a^
	L‐*N*‐methyl‐tyrosine	15^b^	7^b^
	D‐*N*‐methyl‐tyrosine	<1^b^	<1^b^
D315A	*rac‐N*‐methyl‐dopa	48^a^	33^a^
	L‐*N*‐methyl‐dopa	40^a^	28^a^
	L‐*N*‐methyl‐tyrosine	31^b^	27^b^
	D‐*N*‐methyl‐tyrosine	<1^b^	<1^b^

^a^ Ring closed product.

^b^ Demethylated product.

### 3.3 Studying changes in flavin spectra upon substrate conversion

**FIGURE 2:**
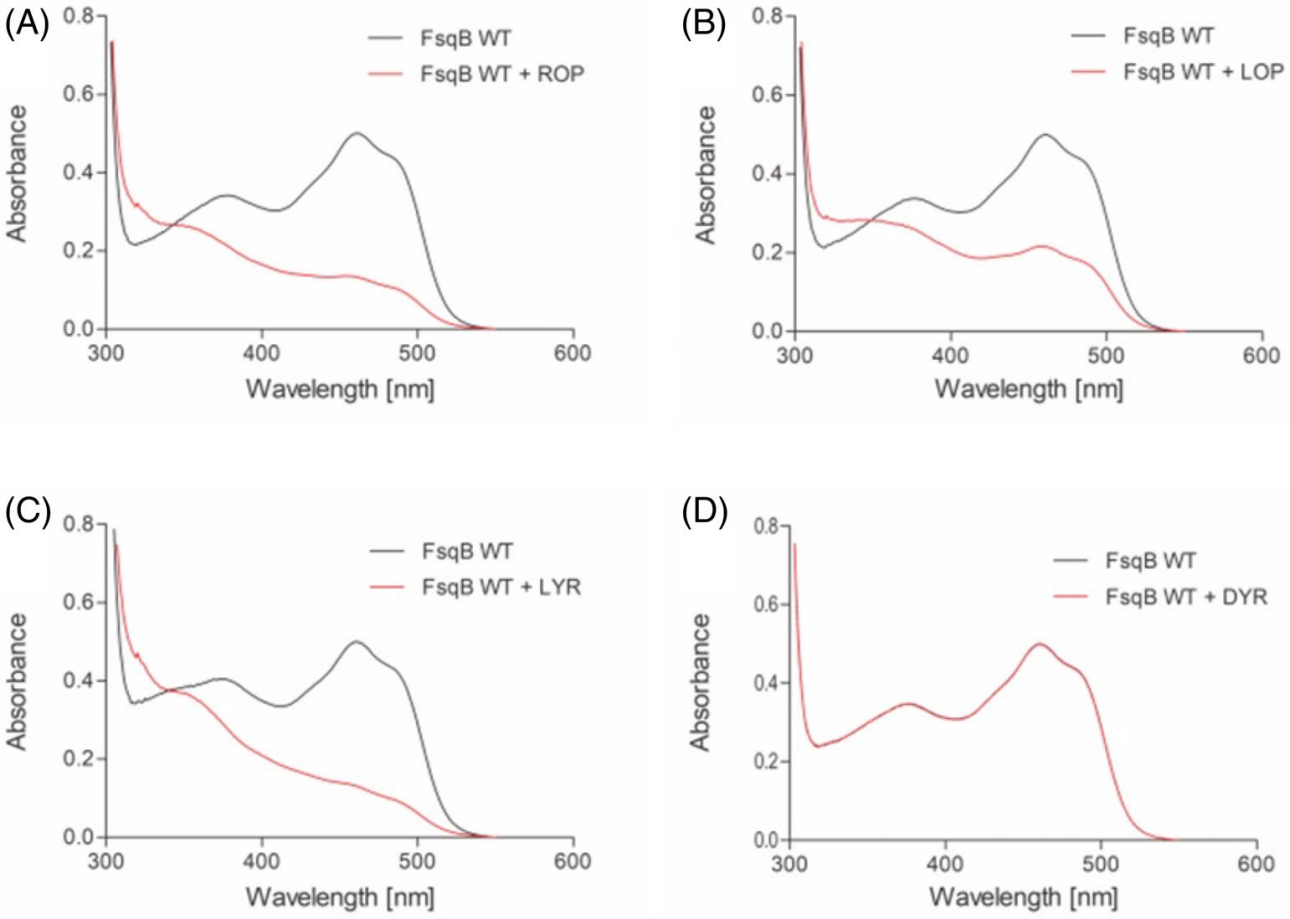
The flavin spectra of FsqB WT with and without the addition of potential substrates. The black lines show the spectra of the oxidized flavin cofactor in FsqB, whereas the red lines show the spectra of the reduced flavin after the addition of racemic *N*‐methyl dopa (ROP) (panel A), L‐N‐methyl dopa (LOP) (panel B), L‐*N*‐methyl tyrosine (LYR) (panel C), or D‐*N*‐methyl tyrosine (DYR) (panel D), respectively. Spectra of FsqB with the respective ligands were recorded immediately after proper mixing of the solution (5 s after the addition of potential substrates).

UV–visible absorption spectra were recorded with FsqB wild‐type and the two variant proteins FsqB Y121F and D315A in presence of three potential substrates ROP, LOP, LYR and the non‐accepted enantiomer DYR. In all experiments, the enzymes were mixed with a 10‐fold excess of potential substrate, and spectra were recorded to follow the changes in flavin spectrum during substrate turnover. Generally, the conversion of the respective substrates with FsqB can be divided into two half‐reactions. In the reductive half‐reaction substrates are oxidized by transferring electrons to the flavin cofactor. In the oxidative half‐reaction, the reduced flavin cofactor of FsqB gets reoxidized by molecular oxygen. Provided that the reoxidation occurs very fast when enough oxygen is available, the reduced form of the cofactor usually cannot be monitored during turnover. However, in our experimental setup high substrate concentrations of at least 600 μM were chosen, which led to the consumption of all oxygen dissolved in the reaction mixtures. Since the diffusion of oxygen into the solution is slower than the reductive half‐reaction of FsqB, an intermediate reduction of the flavin cofactor was monitored and taken as proof for accepted substrates. Reduced flavin spectra were recorded for wild‐type FsqB mixed with an excess of ROP, LOP, and LYR and no spectral changes were found when DYR was added to the enzyme (Figure 2). These results perfectly reflect the findings from the analysis of FsqB‐catalyzed biotransformations. A similar profile was monitored for FsqB D315A, whereas FsqB Y121F showed drastic changes in substrate acceptance. Firstly, only L‐*N*‐methyl tyrosine and not the *N*‐methyl dopa substrates were accepted by this Y121F protein variant. Moreover, the reduction of the flavin cofactor of FsqB Y121F with L‐*N*‐methyl tyrosine was much slower as compared to the wild‐type or D315A protein variant confirming the importance of Y121F for substrate preference.

### 3.4 FsqB binding pose

**FIGURE 3:**
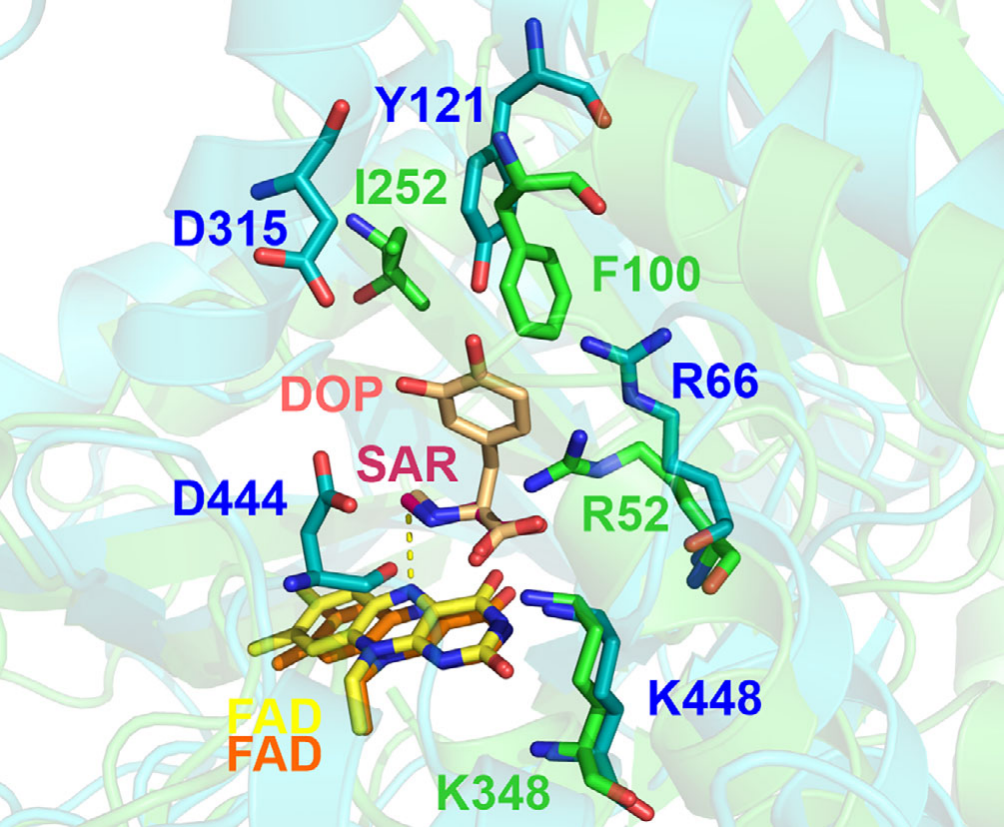
Alignment of the active site of a known ligand‐protein sarcosine oxidase pair, 3QSE (green).^18^ with the crystal structure of FsqB, 6GG2, (cyan).^3^ The distance of the amino group of the ligands to the FAD N5 atom is highlighted with a dashed yellow line. The sarcosine ligand (SAR) of 3QSE is shown in magenta and the starting pose for the D‐*N*‐methyl‐dopa (DOP) in brown. The amino acid labels correspond to the key residues of the binding pocket of FsqB (blue labels for FsqB residues and green labels for sarcosine oxidase residues).

The exact binding pose of the substrates within the FsqB active site has not been determined experimentally yet. In our previous work, we used docking experiments to suggest that *N*‐methyl‐dopa binds in a different orientation than *N*‐methyl‐tyrosine.^3^ However, the suggested docking poses of both enantiomers of *N*‐methyl‐dopa did not correspond to the interactions observed for other members of the SOX family, with the carboxylic group of the ligand pointing towards positively charged residues such as arginine and lysine.^1^ These polar interactions (see Figure 3) are of utmost importance since FsqB only accepts ligands with a carboxylic functional group.

In the current work, we initially generated a binding pose for DOP, by using the structure of sarcosine oxidase with PDB code 3QSE as Reference 18. First, both proteins were superposed using the residues around the active site to ensure a proper alignment of the region of interest. Then, the DOP was fitted to match the orientation of the other known substrates and slightly adjusted to avoid atom clashes. The resulting pose, Figure 3, was stable during all the simulations performed and fulfilled all the known relevant interactions, the interaction of the carboxylic group with the positively charged region of the enzyme, (R66, K448) and a hydrogen bond to D444.^1^, ^3^

### 3.5 Free energy perturbation

To obtain the binding pose of the other ligands and to estimate the strength of binding of the different ligands, free energy perturbations were performed using the extended TI algorithm.^26^ The following perturbations were performed for both the ligand in solvent and bound to the protein: DOP to LOP, DYR, LYR; LOP to L‐*N*‐methyl‐tyrosine and LYR to DYR. Free‐energy differences were obtained for the compounds in solvent and in complex with the FsqB enzyme, such that relative binding free energies, ∆∆G, may be obtained (see Table 3). In Reference 3 Kd values for LOP and the racemic mixture of N‐methyl‐dopa are reported, with only a slight loss of affinity for the racemic mixture. This would suggest a similar binding strength between the two, with a relative binding affinity of maximally +1 kJ/mol, where we find +16 kJ/mol. Note, however, that using a racemic mixture is a rather indirect way to determine the binding affinity of two stereoisomers, and competitive binding cannot be excluded. The obtained free energy perturbations produced overestimated ∆∆G differences when compared to the known *K*_*M*_ values (ROP <0.16 mM, LOP = 0.27 mM, LYR = 2.1 mM, DYR NA).^3^ However, the free energy differences correctly ranked the ligands under study based on their *K*_*M*_ values. This resulted in DOP having a higher binding affinity for FsqB than LOP, followed by LYR, and finally, DYR showcasing the lowest affinity to FsqB. Furthermore, the cycle closure obtained by adding up the free‐energy differences along closed cycles with at least three compounds (e.g., DOP → LOP → LYR → DYR → DOP) had an acceptable range of hysteresis, with a maximum of 2.4 kJ/mol, suggesting that the simulations converged properly, see Table 3. The same behavior can be observed when comparing direct perturbations (e.g., DOP → LYR) to indirect ones (e.g., DOP → LOP → LYR), with hysteresis lower than 2.5 kJ/mol.

**TABLE 3:** Free energy perturbation results in (kJ/mol).

Perturbation^a^	∆G solvent^b^ (kJ/mol)	∆G protein^c^ (kJ/mol)	∆∆G binding^d^ (kJ/mol)
DOP → LOP	−0.6	16.0	16.6
DOP → DYR	−108.0	−77.3	30.7
DOP → LYR	−108.2	−82.3	25.9
LOP → LYR	−108.0	−101.1	6.9
LYR → DYR	−0.8	4.9	5.7

^a^ The ligands perturbed are shown with the following abbreviations: DOP, D‐*N*‐methyl‐dopa; DYR, D‐*N*‐methyl‐tyrosine; LOP, L‐*N*‐methyl‐dopa; LYR, L‐*N*‐methyl‐tyrosine.

^b^ “∆G solvent” shows the free energies obtained from the perturbation of the ligands in solvent.

^c^ “∆G protein” show the free energies obtained for the ligands bound to FsqB.

^d^ “∆∆G binding” is the relative binding free energy.

Notably, the computed free‐energy differences are significantly higher than what one would expect from the *K*_*M*_‐values alone. However, *K*_*M*_‐values are model parameters, that are not strictly representative of the affinity of the compounds. At most, they represent affinity of the catalytically relevant poses, which may be obfuscated in complex mechanisms of action as the one under study. Therefore, making a quantitative comparison between affinity measures (binding free energies) and activity data (*K*_*M*_) impossible. Despite of this, since the affinity is one of the factors that influences the *K*_*M*_‐values it is possible to establish a qualitative correlation. Additionally, the overestimation of the binding free energies could be explained due to inaccuracies in the forcefield, or due to a lack of sampling in the performed simulations.

**FIGURE 4:**
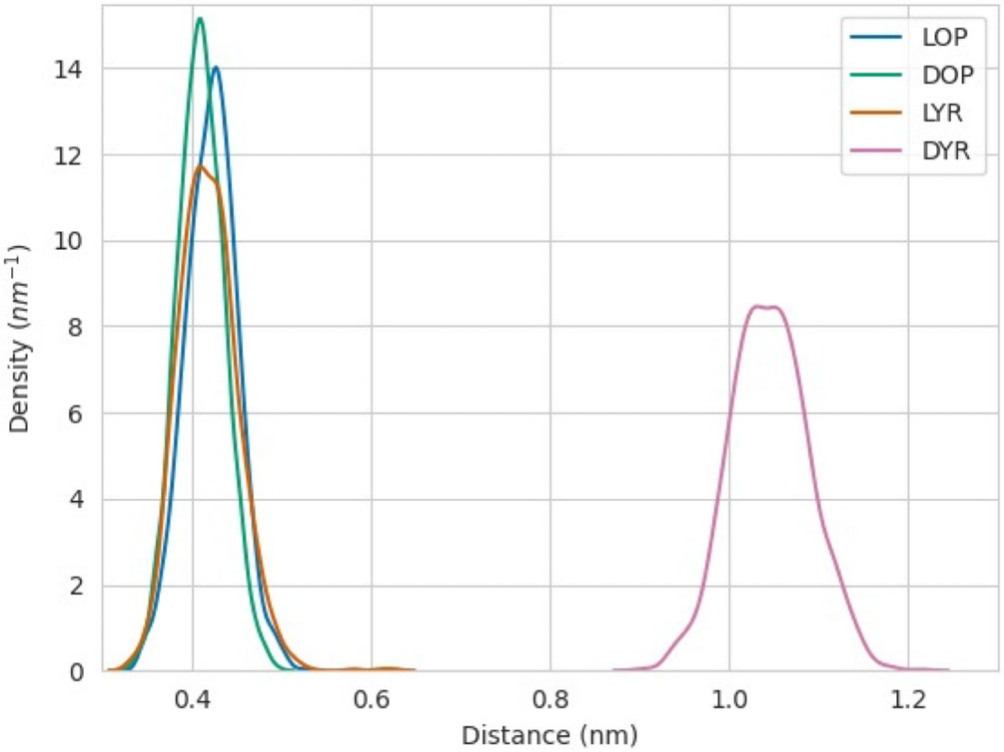
Probability density distribution of the distance of the methyl group of the ligands under study to the N5 atom of the FAD cofactor. The short form for the ligand are as follows: DOP, D‐*N*‐methyl‐dopa; DYR, D‐*N*‐methyl‐tyrosine; LOP, L‐*N*‐methyl‐dopa; LYR, L‐*N*‐methyl‐tyrosine.

The simulations of the final binding poses were elongated for 40 ns to be able to extract dynamic information from them. From the last 20 ns of these simulations of each ligand bound to the protein, the distance from the methyl group of the ligands to the catalytic nitrogen of the FAD cofactor, N5, was monitored (indicated in Figure 3). QM/MM studies performed for the model enzyme sarcosine oxidase revealed that the reaction mechanism involves electron transfer from the methyl group of the substrate to the reactive N5 position of the flavin cofactor.^28^ Since FsqB belongs to the sarcosine oxidase family of enzymes, a minimal distance of the methyl group of the substrate to the N5 atom of the isoalloxazine ring is a prerequisite for catalysis. The distance distributions in Figure 4 reveal that all the compounds that experimentally show reactivity within the FsqB enzyme have a similar distance distribution averaging at 4.0 Å and reaching shorter, catalytically relevant, distances periodically. On the other hand, the ligand DYR, for which no reaction is observed, has a significantly higher distance distribution, never reaching distances smaller than 4.0 Å during the simulation.

**FIGURE 5:**
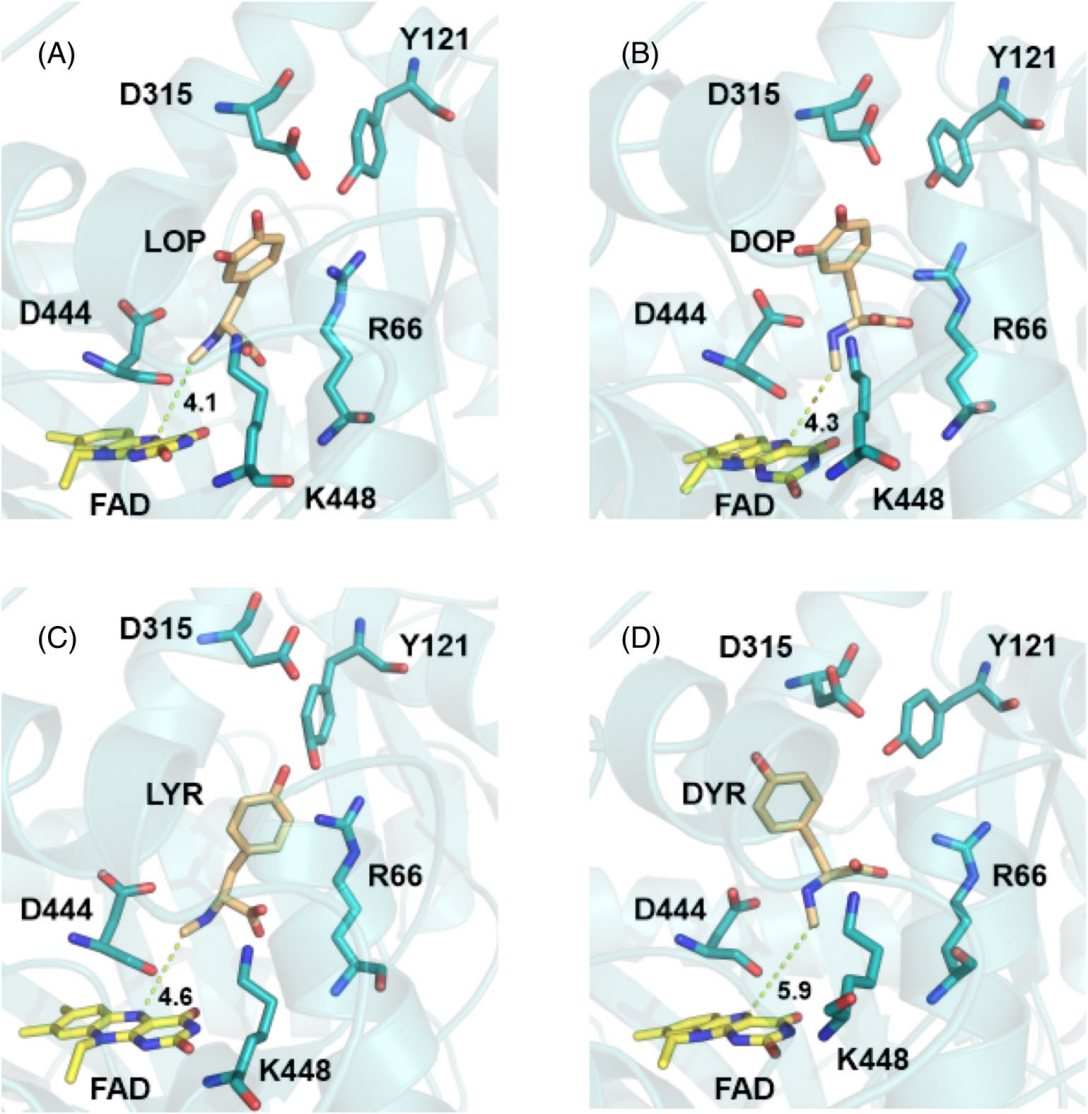
Representative binding poses of the different ligands under study: (A) LOP: L‐*N*‐methyl‐dopa, (B) DOP: D‐*N*‐methyl‐dopa, (C) LYR: L‐*N*‐methyl‐tyrosine, (D) DYR: D‐*N*‐methyl‐tyrosine. Depicted in cyan are the protein residues, in yellow the FAD cofactor, and in brown the ligands. All substrates maintain the interactions with the residues that were reported to be important for sarcosine oxidases.^3^

Apart from the rather unfavorable binding affinity, this difference in distance distribution provides an additional explanation of why this specific compound is not reactive. To further understand the binding capabilities of the different ligands under study, their binding poses were examined (Figure 5). Aside from the already known key interactions (R66, K448, and D444), the reactive ligands displayed hydrogen bonding patterns from the catechol moiety to a negatively charged region located above the FAD cofactor formed by the closely interacting residues, Y121 and D315. Based on the analyses of the binding poses we hypothesize that these residues play an important role in the correct orientation of all the reactive compounds.

### 3.6 Site‐directed mutagenesis study

To validate our hypothesis of the relevance of residues Y121 and D315, we tested experimentally and computationally the two following protein variants, Y121F and D315A. Computationally, the variants were obtained by slowly perturbing the replaced residue using 11 λ‐windows of 1 ns each. The variant proteins were then simulated for 40 additional nanoseconds. The first 20 ns were discarded to allow the system to relax and the remaining 20 ns were examined.

**FIGURE 6:**
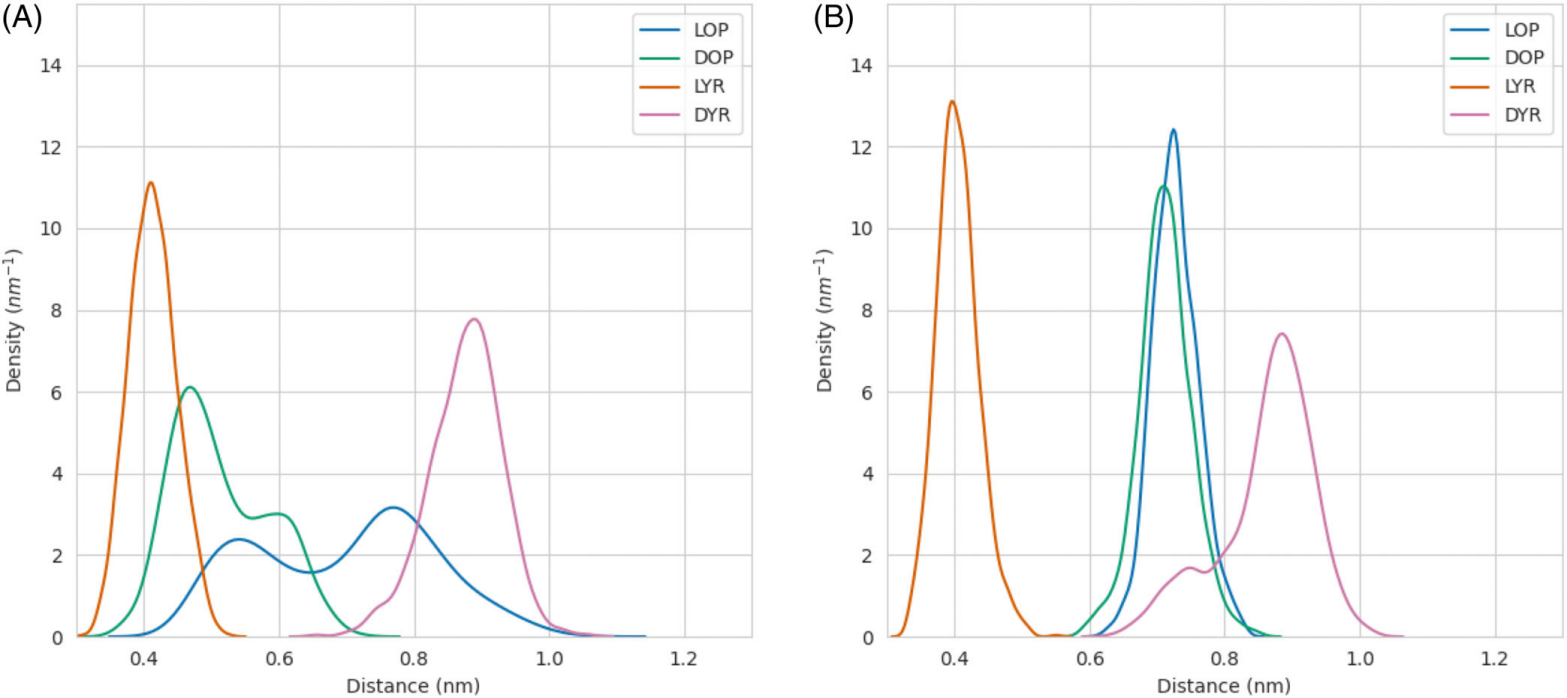
Probability density distribution of the distance of the methyl group of the ligands under study to the N5 atom of the FAD cofactor for the D315A (panel A) and the Y121F variants (panel B). The short form for the ligand are as follows: DOP, D‐*N*‐methyl‐dopa; DYR, D‐*N*‐methyl‐tyrosine; LOP, L‐*N*‐methyl‐dopa; LYR, L‐*N*‐methyl‐tyrosine. The peak at lower distances observed in the case of the D315A variant for the ligands DOP, and LOP, correspond to the ligand distances before the ligand detached.

Both variants led to a detachment of the majority of the bound ligands, which can be observed in their distance distributions to the N5 of the FAD in Figure 6, with the only exception being LYR, which still remained somewhat close to the catalytic distances. Replacement of the residue tyrosine 121 with phenylalanine produced a more sudden perturbation of the binding cavity compared to the amino acid exchange of aspartate 315 to alanine. For the D315A variant, the bound ligands required longer timescales to completely detach as observed in the extra peak of their distance distribution (Figure 6A).

**FIGURE 7:**
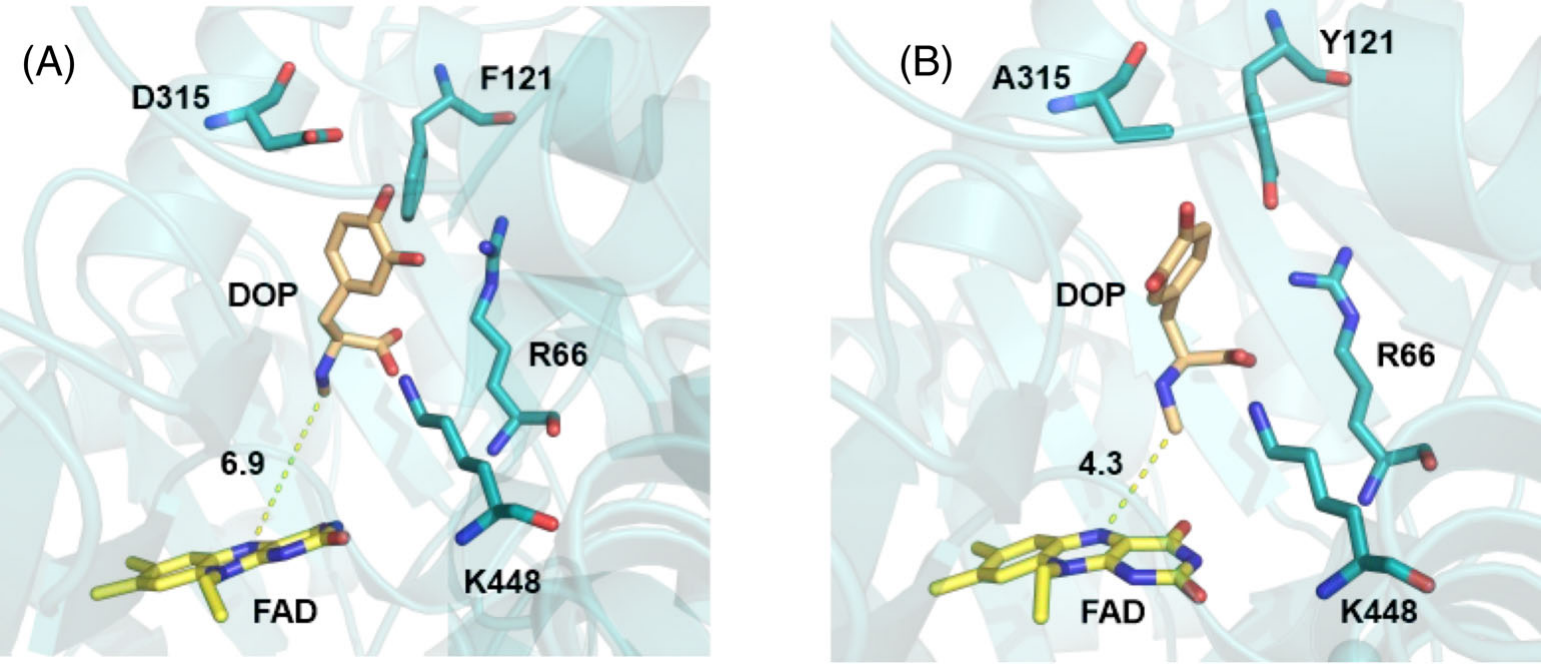
Binding pose perturbation after replacement of residue Y121 to Phe, (panel A), and of D315 to Ala (panel B). Depicted in cyan are the protein residues, in yellow the FAD cofactor and in brown the ligand D‐*N*‐methyl‐dopa (DOP) with the binding orientation of the last configuration after the mutation.

A closer examination of the simulations revealed that for both variants the detachment is produced by a shift in the position of the residues that were constituting the negatively charged region of the catalytic pocket, which pushed the ligands to migrate to interact with other charged residues (Figure 7). A similar interaction occurred for the only ligand that remained at a relatively close distance to the FAD cofactor. Based on the MD data, it is unclear if the ligand will eventually diffuse away or if this different orientation would still be suitable for an enzymatic reaction.

## 4 DISCUSSION

**FIGURE 8:**
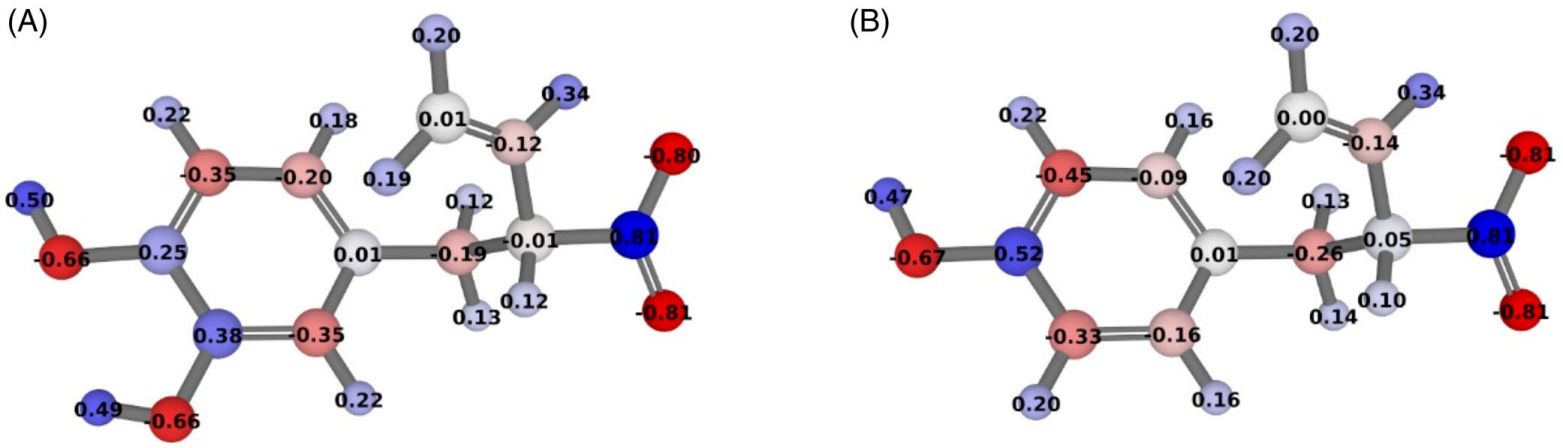
The DFT/ωB97X‐D/6‐311++G(d,p) optimized structures and partial atomic charges of the iminium intermediates of (A) L‐*N*‐methyl‐dopa and (B) L‐*N*‐methyl‐tyrosine. Atoms are colored according to their charge (positive atoms are blue, negatively charged atoms are red).

FsqB is able to bind and accept both *N*‐methyl‐dopa and *N*‐methyl‐tyrosine as substrates, however, it catalyzes a cyclization reaction for both enantiomers of *N*‐methyl‐dopa, whereas for *N*‐methyl‐tyrosine only the L‐form is reactive and results in the formation of the demethylation product, see Figure 1.^3^ As shown in previous studies,^1^, ^3^ the enzyme oxidizes the respective substrate to a short‐living iminium intermediate, see Figure S2. In aqueous environment, the imine group then hydrolyzes spontaneously to yield amine and formaldehyde. Besides that, a concurrent ring closure reaction between the imine group and the phenyl ring is possible. This pathway follows the electrophilic substitution mechanism and is favored by several aspects: (1) The cyclization is intramolecular, that is, fast enough to occur before the imine gets attacked by a water molecule; (2) formation of a stable 6‐membered ring; (3) negative partial charges on the phenyl ring promote the electrophilic attack by the iminium group. The main difference between the substrates is the electron structure of the aromatic ring. In *N*‐methyl‐tyrosine, the mesomeric effect of the *para*‐hydroxy group leads to the accumulation of the π‐electrons in the *meta*‐position. The electrophilic attack on this position would lead to an unfavorable 7‐membered ring, so the hydrolysis path is preferred. However, in *N*‐methyl‐dopa, the additional mesomeric effect of the *meta*‐hydroxyl group pushes the electron distribution into the *ortho*‐position, which can be subsequently attacked by the carbon of the iminium group to form a stable 6‐membered ring. To verify this hypothesis, we analyzed the charge distribution of the iminium intermediates at the DFT/ωB97X‐D/6‐311++G(d,p) level. The partial atomic charges in Figure 8 clearly show the increased electron density in the *ortho*‐position of *N*‐methyl‐dopa compared to *N*‐methyl‐tyrosine, which explains the different behavior of the two substrates during oxidation and thus the ring‐closed and demethylated products, respectively. We also performed a charge assignment on the species with the deprotonated phenol group, the phenolate. The preferred site for the electrophilic attacks remained the same, although with slightly more negative charges. This is in accordance with the known substituent effect of the phenolate group, which is a stronger electrophilic substitution activator to the ortho‐/para‐position than the phenol. We conclude that the phenol group deprotonation is not decisive for the mechanism.

Interestingly, the simulations described in this work again confirmed that FsqB can exceptionally catalyzes the oxidation of both stereoisomers of *N*‐methyl‐dopa although it belongs to the family of SOX, which is well known to accept only the L‐stereoisomers of *N*‐methyl‐amino acids.^1^ On the other hand, FsqB does not accept DYR as substrate. This substrate specificity was already found in wet‐lab experiments and led to the hypothesis that a tyrosine residue, Y121, causes this substrate preference in FsqB (see HPLC results in Table 2). Superimposition of the structures of FsqB (PDB code: 6GG2) and sarcosine oxidase (PDB code: 3QSE) shows that this important tyrosine residue is missing in the model enzyme sarcosine oxidase and that a phenylalanine residue is found instead at the above‐mentioned position (Figure 3). We assume that this Y121 was adapted to fix the ligands by forming hydrogen bonds with hydroxyl groups of the benzene ring (for both *N*‐methyl dopa and *N*‐methyl tyrosine).

**FIGURE 9:**
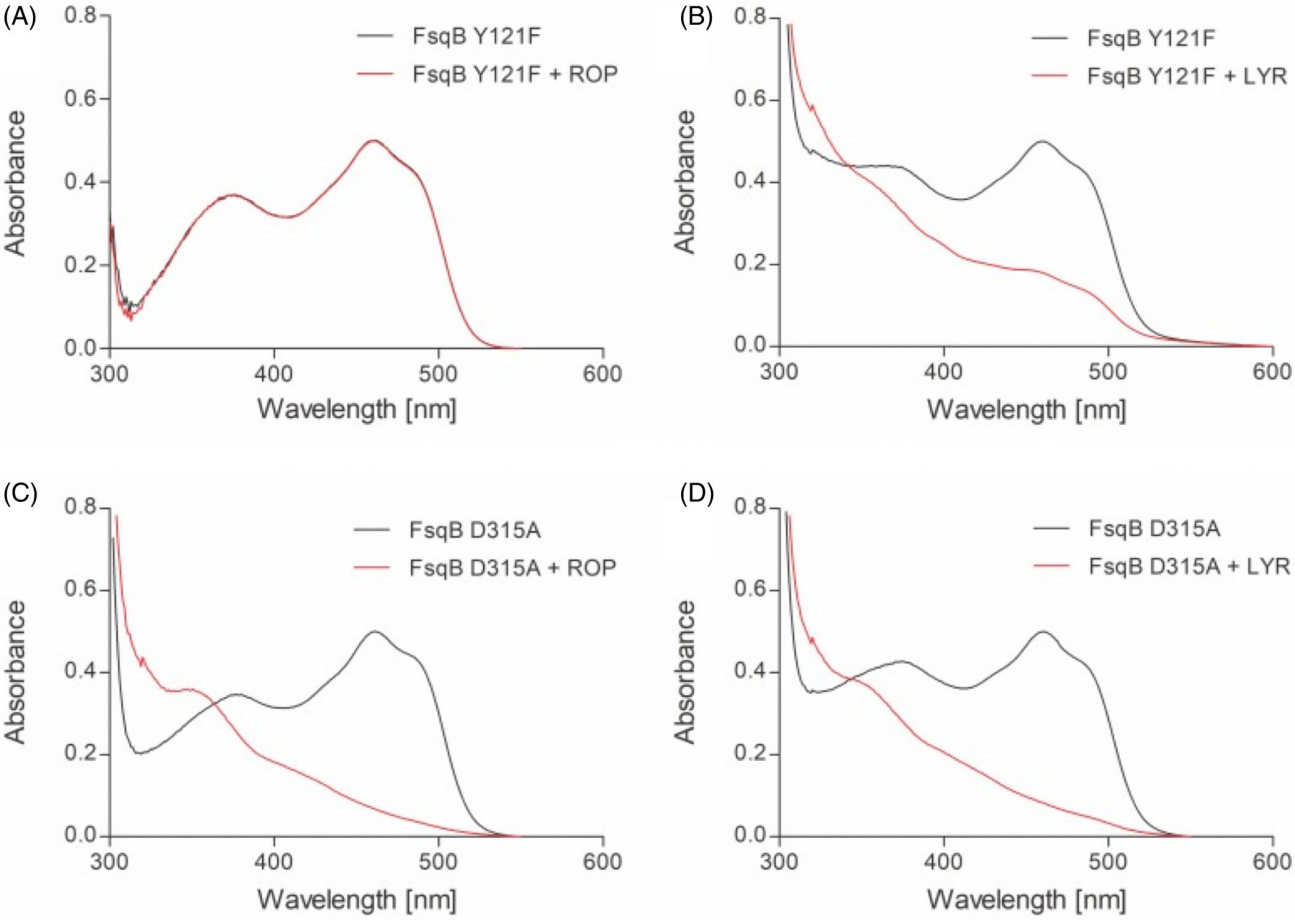
The flavin spectra of FsqB Y121F (panel A, B) and D315A (panel C, D) with and without addition of potential substrates. The black lines show the spectra of the oxidized flavin cofactor in FsqB, whereas the red lines show the spectra of the flavin after the addition of racemic *N*‐methyl dopa (ROP) (panel A, C) or L‐*N*‐methyl tyrosine (LYR) (panel B, D), respectively.

A “typical” substrate for enzymes belonging to the sarcosine oxidase family, stays in 4 Å distance to the catalytically active N5 position of the flavin ring (Figure 4). The molecular simulations performed with the Y121F variant showed that the Y121F substitution causes the studied ligands to move away from the FAD cofactor in the active site, with one exception: LYR. In the Y121F variant, the biotransformation results show a drastic decrease, of more than 6 to 13‐fold, in the capability of FsqB to convert the substrates into their respective products, with a slightly increased product formation for LYR (Table 2). The significant decrease in activity for the Y121F variant goes hand in hand with the observed quick distortion of the active site after Y121 is modified to phenylalanine in the simulations (Figures 6 and 7). Although both LOP and DOP substrates achieve better kinetic rates than LYR in WT FsqB,^3^ the latter ligand seems to bind more stably to the active site of the enzyme in the absence of Y121. The spectral study of the variants showed that ROP did not result in changes in the flavin spectra of Y121F, whereas, LYR was still accepted as substrate and led to cofactor reduction (Figure 9, panels A and B, respectively).

For the D315A variant, the decrease in activity is more modest, around 2‐fold, than in the Y121F variant protein (Figure 7B). Based on the MD simulations of the wild‐type protein, the role of D315, seems to aid Y121 to be in the correct orientation to interact with the substrates. Replacing this residue led to a change in orientation of Y121 and to ligands diffusing from their original predicted binding poses. The spectral study of the variant D315A shows that both ROP and LYR can still reduce the flavin in the D315A variant (Figure 9, panels C and D respectively).

The MD results together with the experimental results seem to suggest that, in the D315A variant, Y121 is still capable of achieving the orientation needed for FsqB to be active, but without D315 restricting its orientation, this catalytical orientation is achieved less often leading to a modest decrease in the observed activity. Our hypothesis is that in the D315A variant, Y121 can fluctuate between the wild‐type orientation and the one observed in the last snapshots of the MD simulation, decreasing its availability to bind the substrate. With the length of the MD simulations performed it is not possible to discriminate if Y121 would eventually recover its catalytical orientation or if there is another mechanism in play. Removing the hydrogen bonding capacity of Y121 entirely in the Y121F variant leads to a much more pronounced loss in activity, confirming its relevance in the orientation of the substrates.

In summary, our experiments and molecular simulations are in close agreement with each other and offer the following explanations to solve the riddles that are posed by Figure 1: D‐*N*‐methyl‐tyrosine binds with the lowest affinity to the active site of FqsB and moves away from the FAD cofactor, explaining why no product formation is observed. Both stereoisomers of *N*‐methyl‐dopa and LYR form a stable and reactive interaction with the active site and are transformed to an imine intermediate. The increased electron density in the *ortho*‐position of *N*‐methyl‐dopa as compared to LYR, however, leads to different product formation: the stereoisomers of *N*‐methyl‐dopa form the intramolecular ring closure, while the LYR leads to demethylation. Furthermore, we have established that Y121 is highly relevant to maintain the catalytically active poses of the substrates, and that D315 in turn helps to orient Y121 in the appropriate orientation. Overall, our interdisciplinary approach could shed light on initially confusing processes of molecular recognition by the FsqB enzyme.

## AUTHOR CONTRIBUTIONS

Oriol Gracia Carmona, and Peter Poliak performed the computational calculations; Majd Lahham, Dominic Goj, Eva Frießer and Silvia Wallner performed the wet‐lab experiments; Peter Macheroux and Chris Oostenbrink supervised the work. Oriol Gracia Carmona, Majd Lahham and Chris Oostenbrink planned the research, Oriol Gracia Carmona and Majd Lahham wrote the first draft of the manuscript, Oriol Gracia Carmona, Majd Lahham, Peter Poliak, Silvia Wallner, Peter Macheroux and Chris Oostenbrink finalized the manuscript. All authors have read and approved the final manuscript.

## CONFLICT OF INTEREST STATEMENT

The authors declare no conflicts of interest.

## Supporting information


**Data S1.** Supporting Information.

**Data S2.** Supporting Information.

## Data Availability

The data that supports the findings of this study are available in the supplementary material of this article.
