# Supplementary material for: Understanding the riddle of amine oxidase flavoenzyme reactivity on the stereoisomers of N‐methyl‐dopa and N‐methyl‐tyrosine
Source: J Mol Recognit. 2023 Nov 15;37(2):e3068. doi: 10.1002/jmr.3068 (PMC11475575; doi:10.1002/jmr.3068)
Supplement: Supplementary file 2 — Data S2. Supporting Information. [file JMR-37-e3068-s001.pdf]

Supplementary Information for:

Understanding the riddle of amine oxidase flavoenzyme reactivity on the stereoisomers of N-methyl-dopa and N-methyl-tyrosine

Oriol Gracia Carmona,[1,†] Majd Lahham,[2,3,†] Peter Poliak,[1] Dominic Goj,[2] Eva Frießer,[2] Silvia Wallner,[2] Peter Macheroux,[2] and Chris Oostenbrink[1,4§]

1. Institute for Molecular Modeling and Simulation, Department of Material Sciences and Process Engineering, University of Natural Resources and Life Sciences, Vienna, Austria
2. Institute of Biochemistry, Graz University of Technology, Graz, Austria
3. Department of Biochemistry and Microbiology, Faculty of Pharmacy, Arab University for Science and Technology, Syria
4. Christian Doppler Laboratory Molecular Informatic in the Biosciences, University of Natural Resources and Life Sciences, Vienna, 1190 Vienna, Austria

[†] these authors contributed equally to this work

[§] Corresponding author: chris.oostenbrink@boku.ac.at

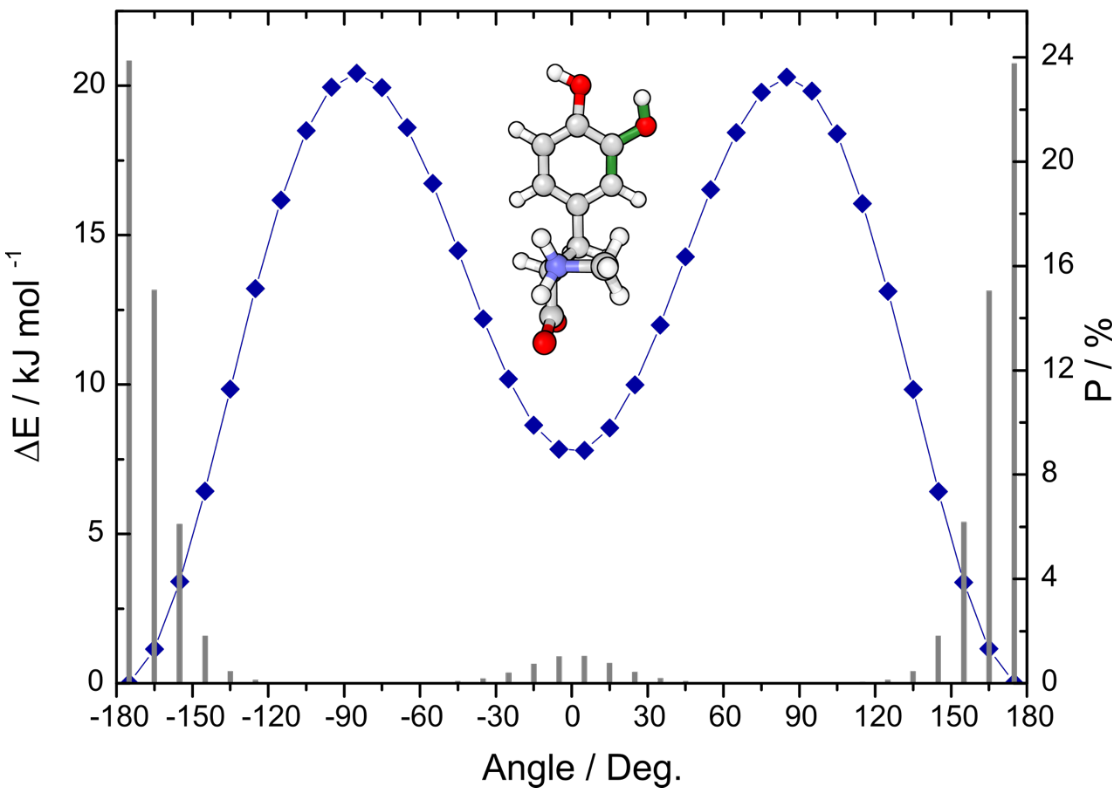

Figure S1: The DFT/B3LYP-D3BJ/6-311++G(d,p) potential energy curve (blue) and the corresponding normalized probabilities according to the Boltzmann distribution (grey) of the *meta*-hydroxyl group dihedral angle in L-N-methyl-dopa (highlighted in green). The curve represents a relaxed scan of the potential energy surface (geometry optimization at every step). Minima were observed at 0° and 180° degrees with the energy difference of 7 kJ/mol separated by the barriers of 21 kJ/mol. The difference can be attributed mainly to the stabilisation by the internal hydrogen bond and the favourable orientation of the oxygen lone pairs. The Lennard-Jones parameters of the hydroxyl groups were added to the forcefield to better reproduce this curve.

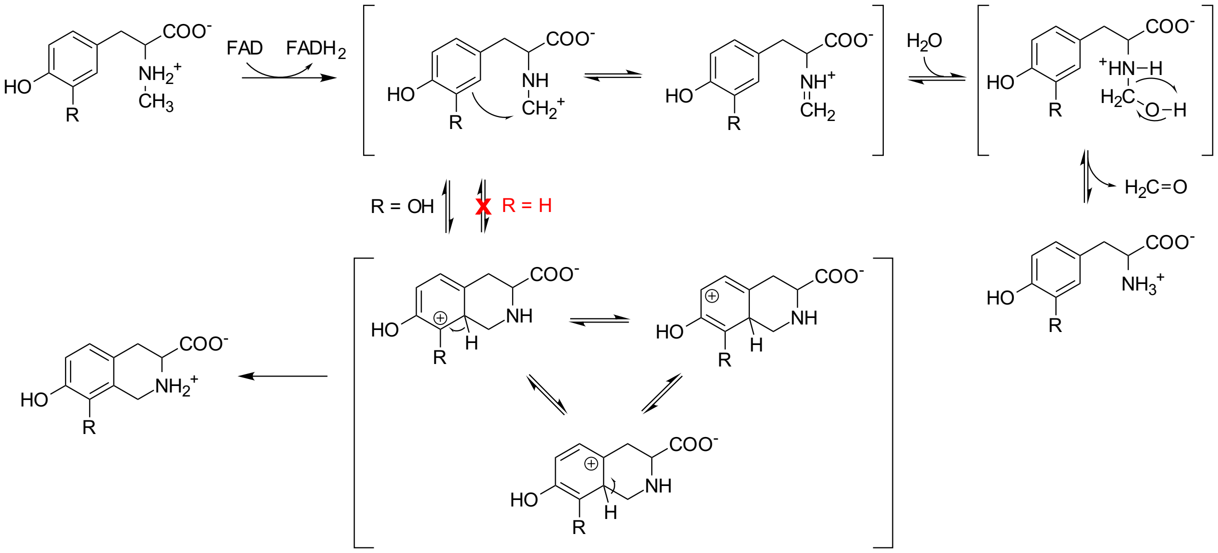

Figure S2: The proposed ring closure mechanism of N-methyl-dopa (R=OH) and N-methyl-tyrosine (R=H) oxidized by FsqB. The square brackets mark the short-living intermediates. FsqB oxidizes N-methyl group to yield the iminium intermediate. Given the sufficient electron density in the *ortho*-position of the benzene ring, the electrophilic iminium group attacks the *ortho*-position leading to a stable 6-membered ring. In the last step, the aromaticity is restored by the deprotonation. If the *ortho*-position does not possess sufficient electron density, as in N-methyl-tyrosine, the iminium group instead hydrolyses into amine and formaldehyde.

Table S1: the primer sequences of the variants Y121F and D315A.

	Forward primer	Reverse primer
Y121F	CCGTGAATTTCATCAAAAAAGCCTAT GCCATTAGCTGCGAACTG	GCTTTTTTGATGAAATTCACGGTTTCCAGTG CTTTCGGAGG
D315A	GGT ATT GTT GCA GTT CTG CGT GAA GCA AGC ATT CAG GTT CCG ACC C	ACG CAG AAC TGC AAC AAT ACC GCT ATA GCT AAA GTG GCC AAA TTT CAG ACA ATT ATC

Table S2: the thermofluor stability results of FsqB WT, the FsqB Y121F, and FsqB D315A

FsqB variant	T_m (°C)
WT	55.0
Y121F	51.5
D315A	56.0
